# Case Report: Necrotizing pulmonary destruction and fatal gangrene: the imperative of early recognition

**DOI:** 10.3389/fmed.2026.1815420

**Published:** 2026-05-13

**Authors:** Tatjana Adzic-Vukicevic, Jelena Jovanovic, Aleksandra Cvetkovic, Maja Zivotic

**Affiliations:** 1 Faculty of Medicine, University of Belgrade, Belgrade, Serbia; 2 Clinic of Pulmonology, University Clinical Center of Serbia, Belgrade, Serbia; 3 Faculty of Medicine, Institute of Pathology, University of Belgrade, Belgrade, Serbia

**Keywords:** *Klebsiella pneumoniae*, necrotizing pneumonia, pulmonary gangrene, pulmonary infarction, pulmonary thromboembolism, secondary infection

## Abstract

**Introduction:**

Pulmonary gangrene is a catastrophic end-stage manifestation of necrotizing pulmonary infection, often arising on the basis of unrecognized and chronically survived pulmonary thromboembolism. Large-vessel thrombosis leads to ischemia and infarction of pulmonary parenchyma, creating an ideal substrate for secondary infection with subsequent rapid tissue devitalization. Impaired perfusion prevents adequate antibiotic delivery, facilitating explosive progression of necrosis.

**Case description:**

We report a fatal case of pulmonary gangrene in a 59-year-old man with a history of chronic alcohol abuse and prior gastric resection due to ulcer disease. Initial imaging demonstrated bilateral cavitations, while clinical deterioration despite broad-spectrum antimicrobial therapy revealed complete destruction of the left lower lobe, hydropneumothorax, and pulmonary embolism. Autopsy confirmed pulmonary gangrene, pulmonary infarction, and bilateral pyothorax.

**Conclusion:**

This case highlights the importance of recognizing pulmonary infarction secondary to thromboembolism as a fertile ground for fulminant infectious destruction.

## Introduction

Pulmonary gangrene represents the terminal phase of lung parenchyma necrosis and is associated with extremely high mortality (1). It may develop when pulmonary infarction, most commonly caused by thromboembolism, becomes secondarily infected (2). Ischemic lung tissue rapidly loses structural integrity, while compromised vascular perfusion prevents effective antibiotic penetration. Consequently, infection spreads explosively through devitalized regions. Early recognition of pulmonary infarction and timely treatment of pulmonary embolism are therefore essential to prevent secondary superinfection and gangrene (3, 4).

## Case description

**Figure 1:**
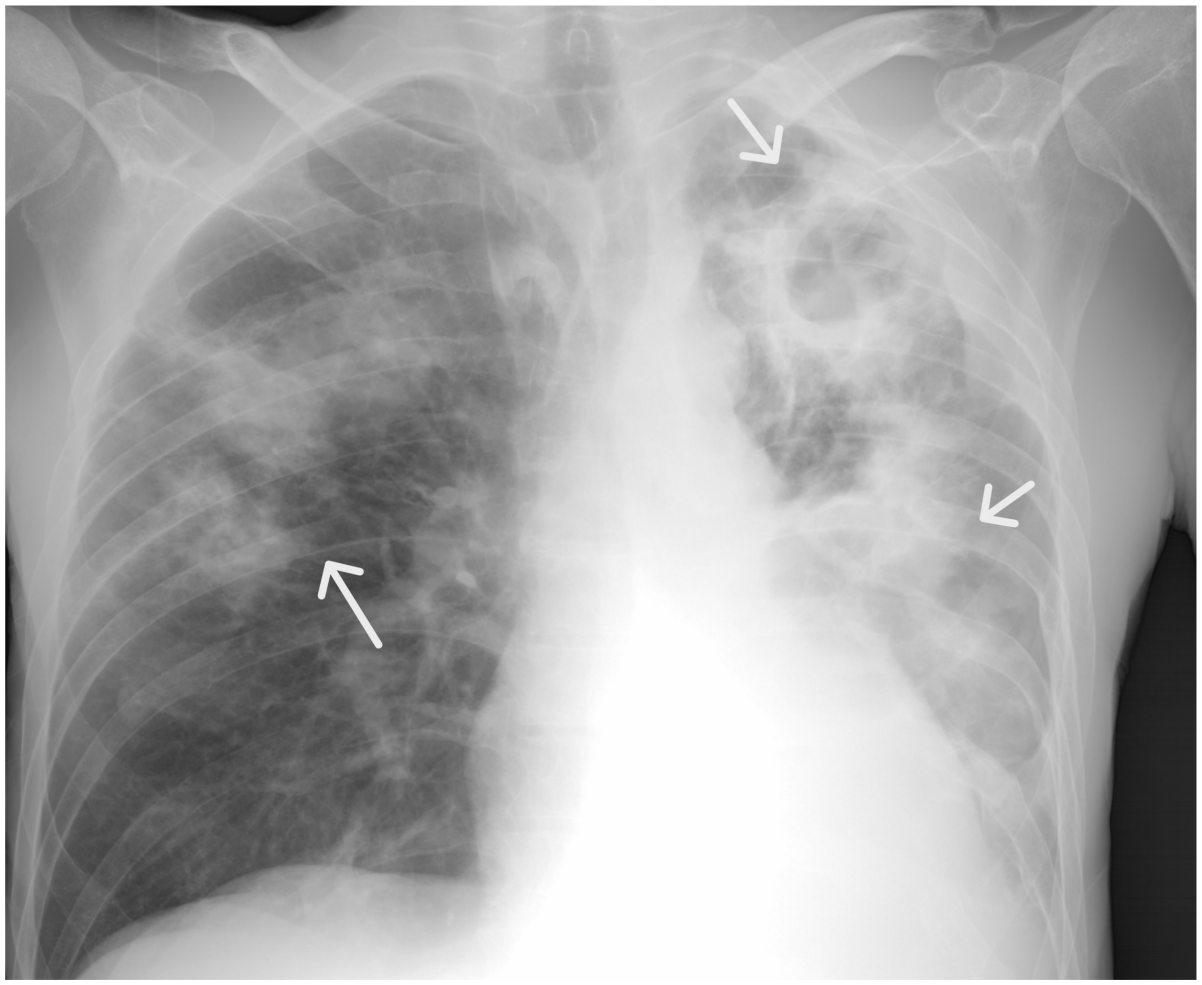
Chest X-ray (anterior–posterior position): Bilateral excavated lung lesions.

We report the case of a 59-year-old man with a history of chronic alcohol abuse and gastric resection who was admitted in severely compromised condition, presenting with months-long fatigue, dry cough, weight loss, and anorexia. Physical examination showed cachexia, pallor, an elevated body temperature of 38.6 °C, hypotension of 110/60 mmHg, a heart rate of 100 beats/min, a respiratory rate of 20 breaths/min, oxygen saturation (SpO2) of 95%, and decreased breath sounds bilaterally. Chest imaging revealed bilateral cavitary lesions (Figure 1). His clinical presentation, including radiography, initially suggested a chronic infectious process. Laboratory evaluation showed elevated inflammatory markers [C-reactive protein (CRP) 183 mg/L, procalcitonin (PCT) 2.1 mg/L, D-dimer 3.6 μg/mL] and significant anemia with a hemoglobin level of 78 g/L. Computed tomography pulmonary angiography (CTPA) identified a right lower lobar pulmonary embolism and extensive destruction of the left lower lobe consistent with pulmonary infarction complicated by superinfection. Bilateral pleural effusions were also present.

Bronchoscopy yielded purulent secretions positive for *Klebsiella pneumoniae* and *Candida albicans*. Blood cultures taken twice were positive for *Klebsiella pneumoniae*. The susceptibility testing confirmed an extensively resistant *K. pneumoniae* strain susceptible only to ceftazidime/avibactam. Acid-fast bacilli (AFB) were not detected, nor were they in the Löwenstein–Jensen cultures we got later. Human immunodeficiency virus (HIV), hepatitis B antigen (HBsAg), and anti-hepatitis C virus (HCV) were negative, while 1,3-β-D-glucan was positive. Treatment started with meropenem, vancomycin, metronidazole, antifungals including micafungin and voriconazole, and low-molecular-weight heparin (LMWH) in a therapy dose for pulmonary embolism according to anti-Xa level.

**Figure 2:**
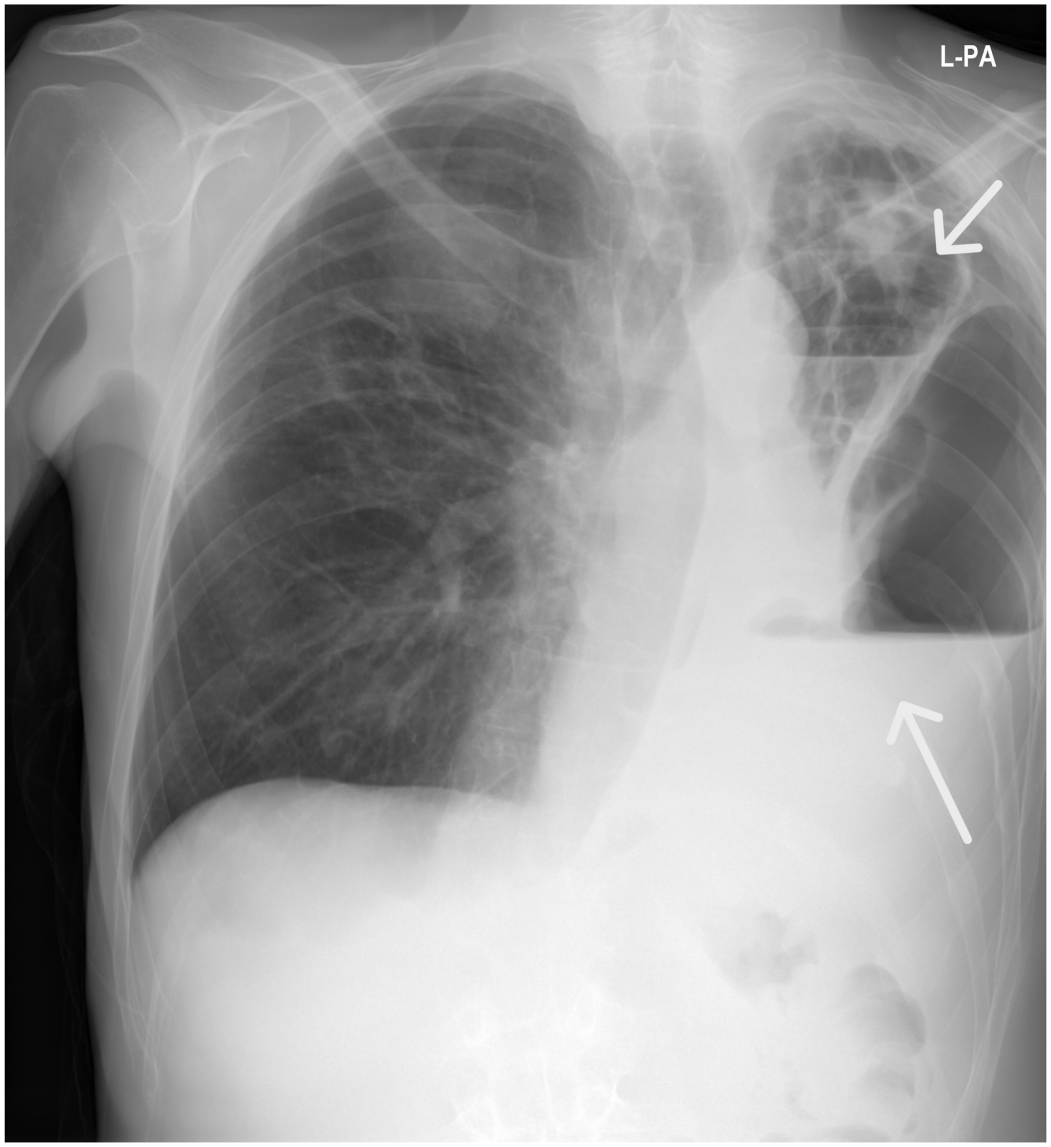
Chest X-ray (posterior–anterior position): Bilateral excavated lung lesions with left-sided hydropneumothorax.

**Figure 3:**
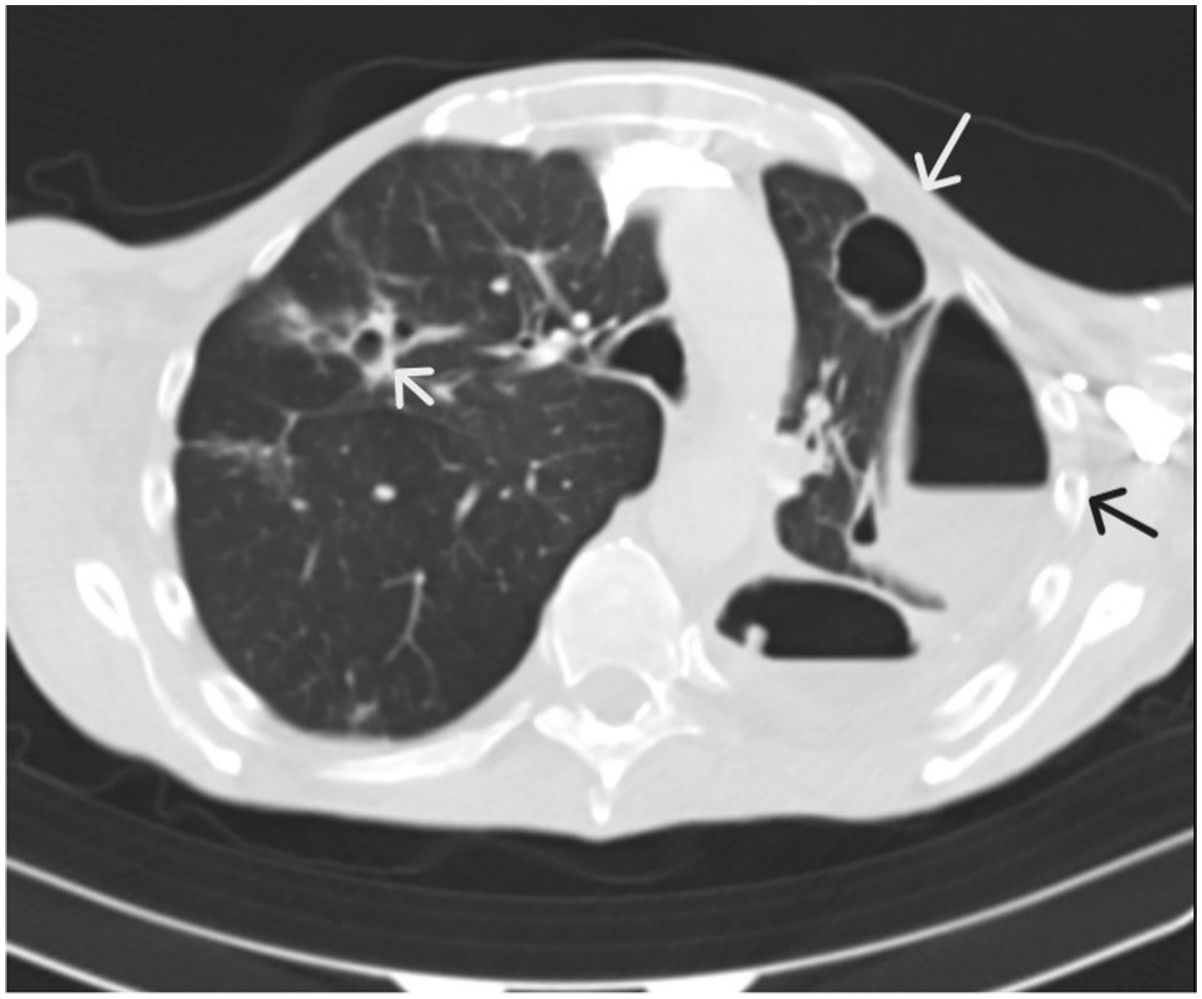
Lung computed tomography with bilateral excavations and left-sided hydropneumothorax.

**Figure 4:**
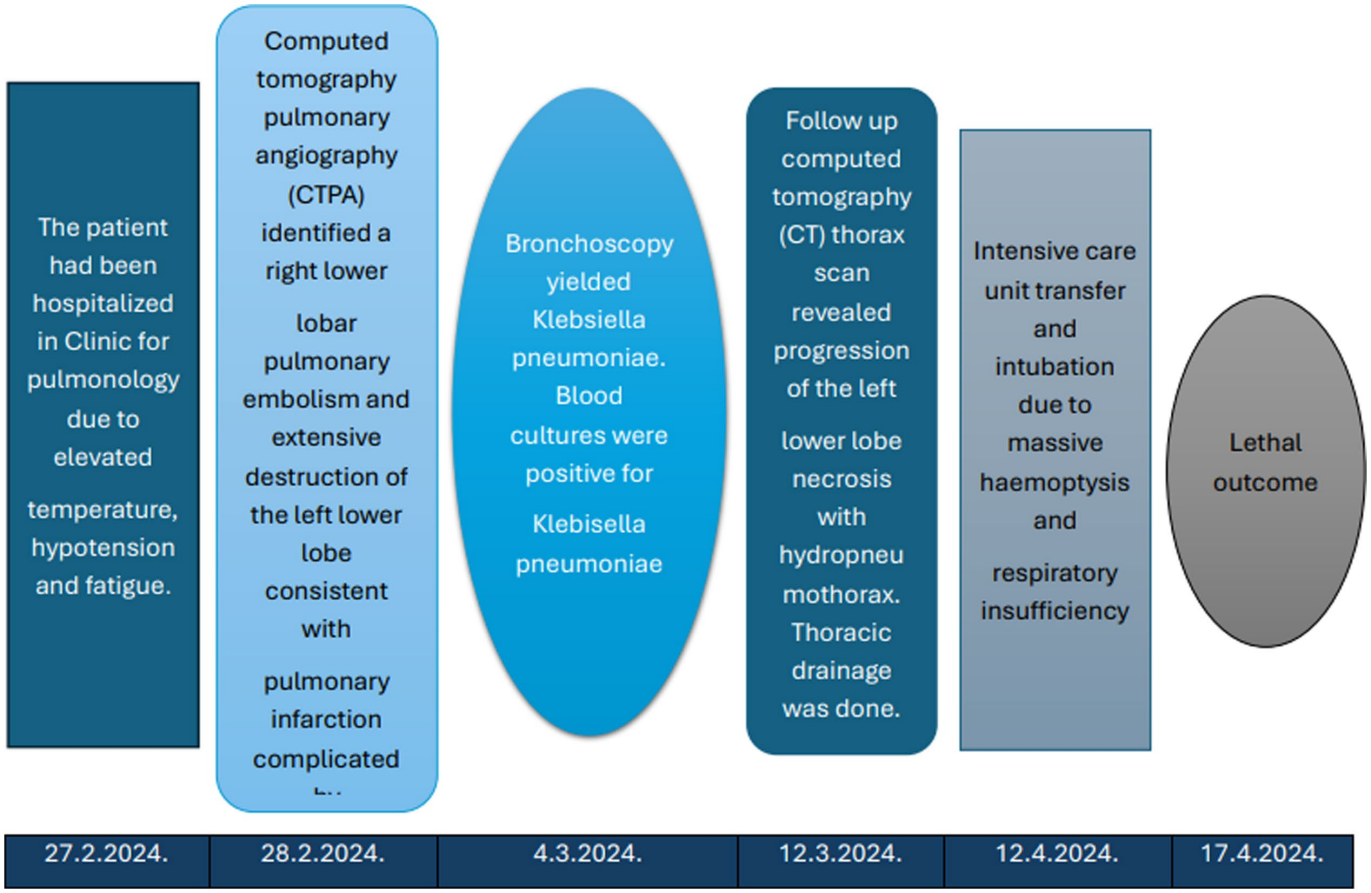
Timeline of the clinical course.

**Figure 5:**
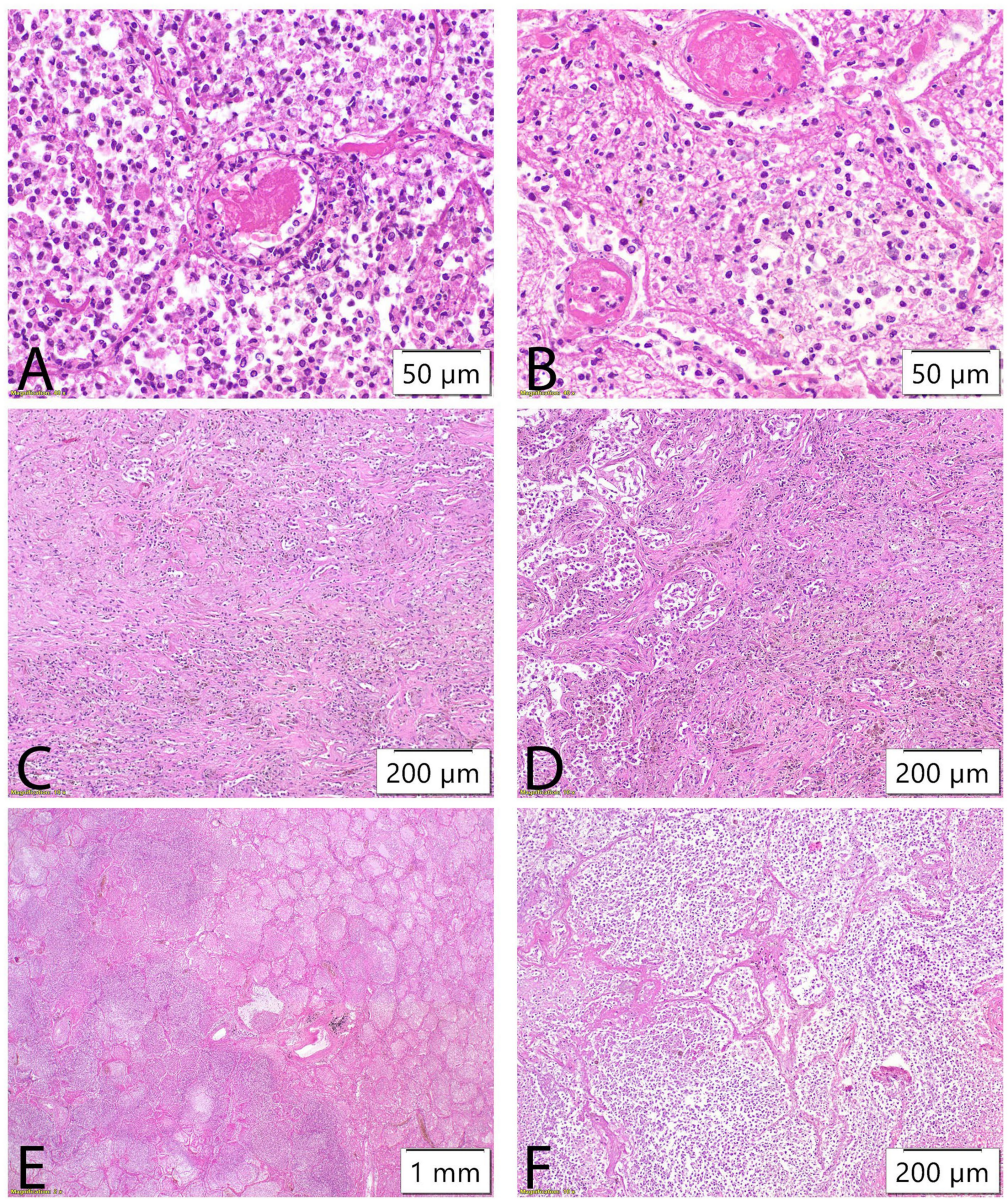
Morphology of pulmonary gangrene. **(A,B)** The presence of multiple thromboemboli within segmental and subsegmental pulmonary arteries. **(C,D)** Regions of organizing granulation tissue rich in hemosiderophages. **(E,F)** Extensive areas of coagulative necrosis with dense neutrophilic infiltration, morphologically consistent with pulmonary gangrene.

Follow-up CT due to clinical deterioration showed persistent necrosis of the left lower lobe, new necrotic cavities in the right upper lobe, and development of a hydropneumothorax on the left side (Figures 2, 3). Chest tube placement evacuated 1,200 mL of purulent fluid containing Klebsiella spp. and Enterococcus spp. Broad-spectrum antimicrobial therapy with ceftazidime/avibactam, ertapenem, and linezolid continued. Despite continuous drainage, radiologic monitoring, and maximal medical therapy, the patient experienced rapid decline. Clinical deterioration was noticed after 46 days of hospital admission due to hemoptysis. The patient had visible cyanosis with partial oxygen pressure (pO2) of 4.6 kPa and respiratory acidosis pH of 7.14. Owing to developed hemodynamic collapse with hypotension of 60/40 mmHg, the patient required intensive care unit (ICU) transfer, intubation, vasopressors, and cardiopulmonary reanimation (CPR). Repeated bronchoscopy revealed severe airway bleeding. A subsequent cardiac arrest proved fatal, and an autopsy was performed (Figure 4). On both lung cut surfaces, extensive consolidation was present with obstructed bronchi and multiple abscesses. Gross and microscopic examination of both lungs revealed multiple thromboemboli within segmental and subsegmental branches of the pulmonary arteries (Figures 5A,B), some already entering the phase of organization. The pulmonary parenchyma showed sequelae of hemorrhagic pulmonary infarction of varying age and morphology. In several foci, early fibrovascular granulation tissue was present, indicating ongoing organization of infarcted areas, accompanied by numerous hemosiderophages (Figures 1C,D). However, the predominant lesions consisted of extensive areas of coagulative necrosis infiltrated by sheets of neutrophils, representing the morphologic substrate of advanced pulmonary gangrene (Figures 1E,F).

## Discussion

Pulmonary gangrene is an uncommon but devastating condition that frequently arises when pulmonary infarction becomes secondarily infected (1). Although lung abscesses, necrotizing pneumonia, and pulmonary gangrene have a similar pathological process, differences persist in radiological findings, clinical presentation, and causative pathogens (3, 4). Typically, a lung abscess manifests as a slow clinical presentation with a single cavitary lesion. In contrast, necrotizing pneumonia exhibits severe clinical illness and sepsis at the time of presentation (5). Imaging findings include multiple micro-abscesses with more extensive lung parenchyma involvement. Pulmonary gangrene is characterized by a greater extent of necrosis, more than 50% of the affected lobe, and larger vessel thrombosis compared to necrotizing pneumonia (6). Radiologic hallmarks in pulmonary gangrene include multiple cavitations that may coalesce into a large cavity, hydropneumothorax, and the air crescent sign (7). It is considered that more than 20% of patients with necrotizing lung disease develop pneumothorax. The relatively high prevalence of pneumothoraces in the setting of necrotizing disease in critically ill patients can be attributed to barotrauma due to a high level of ventilator pressure in combination with the underlying pathology of lung necrosis and the development of cavitation (8). However, CT remains the most sensitive modality for assessing disease extent and identifying vascular thrombosis (5–8).

Thromboembolism causes occlusion of segmental or lobar pulmonary arteries, leading to ischemia and infarction. Infarcted tissue, devoid of perfusion, is highly susceptible to bacterial colonization, particularly by organisms such as *Klebsiella pneumoniae*, which is prevalent among chronic alcohol users (9). Another form of infection that causes necrotizing pulmonary disease is septic pulmonary emboli. In most instances, the source of the septic pulmonary emboli is the heart, especially in the form of tricuspid valve infective endocarditis (10). Other endogenous sources contributing to septic emboli include abscesses, phlebitis, and oral infection, or exogenous sources such as infected intravenous lines (11). Numerous clinical risk factors have been identified in the development of necrotizing pulmonary destruction, including pulmonary gangrene. For instance, influenza co-infection has been a major risk factor for developing necrotizing pneumonia due to impaired macrophage activity (12). Similarly, neutropenic patients experience impaired phagocytic response, predisposing them to necrotizing pulmonary disease with a high mortality rate (13). Other risk factors responsible for pulmonary gangrene include smoking, diabetes, liver disease, and excessive alcohol use (14). Alcohol-related immunosuppression, impaired mucosal defenses, and increased oropharyngeal colonization further predispose to infection, as was seen in our patient case (15).

Once superinfection develops within infarcted regions, the absence of vascular supply prevents antibiotic penetration, resulting in unchecked necrosis. Necrotizing pulmonary disease is most commonly caused by *Streptococcus pneumoniae*, *Staphylococcus aureus,* and *Klebsiella pneumoniae* (2). Other less commonly described bacteria are *Escherichia coli*, *Acinetobacter baumannii,* anaerobes, and, rarely, *Pseudomonas aeruginosa* (16).

Management requires rapid initiation of broad-spectrum antimicrobial therapy and identification of pathogens via sputum, bronchoscopy, or pleural fluid. Nevertheless, once gangrene is established, surgical intervention may represent the only potentially lifesaving option. The role and timing of surgery in managing necrotizing lung disease are highly controversial (17). Surgery should be considered when a patient fails medical treatment, in the case of significant hemoptysis, or in evidence of extensive gangrene (18). Surgical interventions vary from cavitary debridement to lobectomy, both associated with high mortality (19). In the cases of parapneumonic effusions, including empyema, drainage via a chest tube is essential. Additionally, in patients with advanced empyema, video-assisted thoracoscopic surgery (VATS) and/or decortication is indicated (20). Despite aggressive therapy, mortality remains exceedingly high at 45%, especially when diagnosis is delayed (5, 6). Previously reported case series noted a high mortality rate (two of five patients), both intubated with thoracic drain placement, in which one bilobectomy and decortication were done (2). Severe airway bleeding was found to be the clinical manifestation with the highest correlation to death, as was noted in our patient case.

## Conclusion

Pulmonary gangrene is a catastrophic complication that frequently originates from unrecognized pulmonary infarction due to silent or chronically survived pulmonary thromboembolism. Infarcted tissue provides a fertile environment for secondary infection, particularly in immunocompromised or alcohol-dependent patients. Early identification of pulmonary embolism, recognition of ischemic lung injury, and timely initiation of targeted therapy are essential to prevent progression to gangrene. Once necrosis becomes extensive and perfusion is lost, antimicrobial therapy is unable to halt disease progression, leading to rapid clinical deterioration and extremely high mortality. This case underscores the need for heightened clinical vigilance, prompt radiologic evaluation, and early multidisciplinary management to improve outcomes in patients at risk of pulmonary gangrene.

## Data Availability

The raw data supporting the conclusions of this article will be made available by the authors, without undue reservation.
