# Supplementary figures and images for: Case Report: Necrotizing pulmonary destruction and fatal gangrene: the imperative of early recognition
Source: Front Med (Lausanne). 2026 May 13;13:1815420. doi: 10.3389/fmed.2026.1815420 (PMC13212097; doi:10.3389/fmed.2026.1815420)

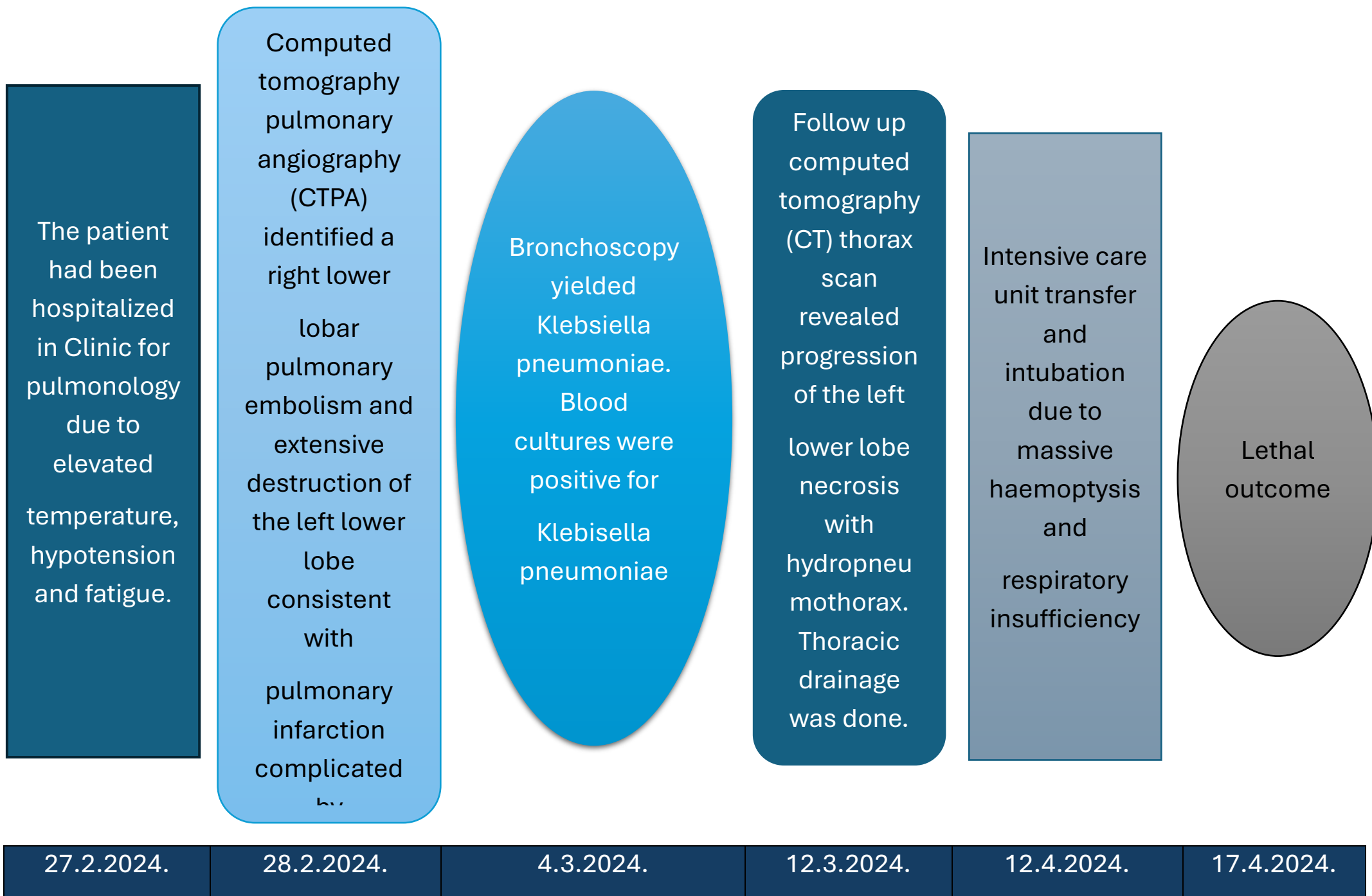

Supplement: Supplementary file 1 [file Data_Sheet_1.pdf]
